# Changes in Walking Speed After High-Intensity Treadmill Training Are Independent of Changes in Spatiotemporal Symmetry After Stroke

**DOI:** 10.3389/fneur.2021.647338

**Published:** 2021-04-01

**Authors:** Brice Cleland, Sangeetha Madhavan

**Affiliations:** Brain Plasticity Lab, Department of Physical Therapy, College of Applied Health Sciences, University of Illinois at Chicago, Chicago, IL, United States

**Keywords:** locomotion (MeSH), high-intensity interval training, spatiotemporal analysis, walking speed, symmetry, stroke

## Abstract

**Objectives:** Decreased walking speeds and spatiotemporal asymmetry both occur after stroke, but it is unclear whether and how they are related. It is also unclear whether rehabilitation-induced improvements in walking speed are associated with improvements in symmetry or greater asymmetry. High-intensity speed-based treadmill training (HISTT) is a recent rehabilitative strategy whose effects on symmetry are unclear. The purpose of this study was to: (1) assess whether walking speed is cross-sectionally associated with spatiotemporal symmetry in chronic stroke, (2) determine whether HISTT leads to changes in the spatiotemporal symmetry of walking, and (3) evaluate whether HISTT-induced changes in walking speed are associated with changes in spatiotemporal symmetry.

**Methods:** Eighty-one participants with chronic stroke performed 4 weeks of HISTT. At pre, post, and 3-month follow-up assessments, comfortable and maximal walking speed were measured with the 10-meter walk test, and spatiotemporal characteristics of walking were measured with the GAITRite mat. Step length and swing time were expressed as symmetry ratios (paretic/non-paretic). Changes in walking speed and symmetry were calculated and the association was determined.

**Results:** At pre-assessment, step length and swing time asymmetries were present (*p* < 0.001). Greater temporal symmetry was associated with faster walking speeds (*p* ≤ 0.001). After HISTT, walking speeds increased from pre-assessment to post-assessment and follow-up (*p* ≤ 0.002). There were no changes in spatiotemporal symmetry (*p* ≥ 0.10). Change in walking speed was not associated with change in spatial or temporal symmetry from pre- to post-assessment or from post-assessment to follow-up (R^2^ ≤ 0.01, *p* ≥ 0.37).

**Conclusions:** HISTT improves walking speed but does not systematically improve or worsen spatiotemporal symmetry. Clinicians may need to pair walking interventions like HISTT with another intervention designed to improve walking symmetry simultaneously. The cross-sectional relation between temporal symmetry and walking speed may be mediated by other factors, and not be causative.

## Introduction

Generally, walking speed is slower in individuals with stroke and is insufficient for safe community ambulation (1, 2), although specific functional effects depend on the individual. Spatial and temporal asymmetries in walking are also common after stroke. Stroke survivors generally have longer step lengths and swing times and shorter stance times in the paretic limb than the non-paretic limb and controls, although there is considerable inter-individual variability (3–9). It is perceived that spatiotemporal asymmetries occur to retain balance amid impaired paretic support and propulsion (1, 7).

Despite the co-occurrence of decreased walking speeds and spatiotemporal asymmetry, it is unclear whether and how walking speed and symmetry are related after stroke. Cross-sectionally, some studies have found that faster walking speeds are associated with greater spatiotemporal symmetry (depending on the measure), while other studies have not found an association (3–8, 10, 11). It is also unclear whether rehabilitation-induced improvements in walking speed are associated with improvements in symmetry (recovery) or greater asymmetry (compensation) (12). It is important to clarify the relation between changes in walking speed and symmetry because many traditional rehabilitative approaches promote spatiotemporal symmetry as a way to facilitate improvements in walking speed, but this strategy may not be necessary (or optimal) to improve walking speed. For example, an individual's range of walking speeds is not associated with spatiotemporal symmetry after stroke (5), and stroke survivors can increase from comfortable to maximal walking speed through a variety of strategies (10, 13, 14).

High-intensity speed-based treadmill training (HISTT) is one rehabilitative strategy that is growing in popularity in the post-stroke population. In individuals with chronic stroke, HISTT leads to improvements in walking speed (15–17) that exceed progressive treadmill training with less training time. Understanding the effect of HISTT on symmetry is important because it may have bearing on its effectiveness and long-term consequences. Some have suggested that high-intensity walking interventions after stroke may require bracing, enhance existing asymmetries, or rely on compensatory strategies to induce improvements in walking speed (18). If HISTT increases walking speed by enhancing walking asymmetry, it may have long term consequences such as limited recovery, overuse injuries, and increased risk of falls (19, 20). A recent meta-analysis indicated that there was no effect of high-intensity treadmill training on symmetry (21). However, data related to spatiotemporal symmetry came from just 4 studies (22–25), which used different exercise modes and intensities, included different levels of stroke chronicity, and different symmetry outcome metrics (temporal and spatial). Furthermore, there was a trend for an overall improvement in symmetry (Z = 1.9, *p* = 0.06). Therefore, it is still not clear what effect high intensity interventions have on spatiotemporal walking symmetry.

The purpose of this study was to: (1) assess whether walking speed is cross-sectionally associated with spatiotemporal symmetry in chronic stroke, (2) determine whether changes in the spatiotemporal symmetry of walking occur after HISTT, and (3) evaluate whether changes in walking speed are associated with changes in spatiotemporal symmetry after HISTT. We hypothesized that faster walking speeds would be associated with greater spatiotemporal symmetry, that spatiotemporal symmetry would improve after HISTT, and that increases in walking speed would be associated with improvements in symmetry.

## Materials and Methods

This study was approved by the institutional review board at the University of Illinois at Chicago (UIC), and all participants provided written informed consent. Data were collected as part of a randomized controlled trial evaluating motor priming and treadmill training (clinical trial registration on ClinicalTrials.gov: NCT03492229) from 2014 to 2018. Deidentified data that underlie study results will be shared by the corresponding author upon reasonable request from qualified investigators immediately following publication.

Eighty-one participants with a single, monohemispheric stroke (chronic: >6 months prior) were enrolled. Inclusion criteria included: age of 40–80 years, residual gait deficits but the ability to walk without external aid for at least 5 min, at least 5° of active paretic dorsiflexion, and a Mini-Mental State Examination score of >21. Participants were excluded if they had brainstem or cerebellar lesions, a Modified Ashworth Scale score of ≥2, major cardiorespiratory or metabolic diseases, or contraindications to transcranial magnetic stimulation (e.g., history of seizures, implanted metallic objects, and use of medications that alter cortical excitability).

The training protocol has been described in detail previously (17, 26). Briefly, participants were assigned to one of four parallel groups receiving 15 min of transcranial direct current stimulation (tDCS; stimulation-based priming), ankle motor tracking (AMT; movement-based priming), tDCS and AMT, or rest immediately prior to HISTT described below. All participants performed 12 HISTT sessions (4 weeks, 3 sessions/week, 40 min/session) after priming interventions. Warmup and cooldown consisted of 5 min of walking at a comfortable pace (50% of weekly tested maximal walking speed). After warmup, intervals of fast walking were performed. For each interval, treadmill speed was increased over a 2 min period up to peak speed, which was held for 10 s. After each interval, participants walked at their warm-up, recovery speed until their heart rate returned within 5 beats per minute of its level during warmup. Throughout the 4-week training period, peak treadmill speed was increased by 10% every subsequent interval if the participant could safely maintain the peak speed achieved during an interval. If the participant could not safely maintain the peak speed, treadmill speed was decreased by 10% for the subsequent interval. Throughout HISTT sessions, participants wore a safety harness without body-weight support and were allowed to hold onto handrails. Manual assistance with hip and knee flexion at toe off was provided as needed. Initial peak walking speed was determined from the maximal walking speed from a 10-meter walk test.

Outcome measures were assessed at pre- and post-assessments, performed within 2 days of the first/last training session and at a follow-up, performed ~3 months after the end of training. At each assessment, walking speed and spatiotemporal characteristics were measured. Walking speed was measured with the 10-meter walk test. Participants performed two trials each at comfortable and maximal walking speeds. Instructions were to walk at a “comfortable” speed or “as fast as safely possible,” respectively. Walking tests were performed without assistive devices whenever possible and use of assistive devices was consistent between timepoints. Time to complete trials was recorded with a stopwatch, and walking speed was computed from the mean across trials. Spatiotemporal characteristics of walking were measured during walking trials across the GAITRite electronic walkway [classic 14' (4.27 m) model, CIR Systems Inc., NJ, USA]. Participants performed two trials each at their comfortable and maximal walking speeds. Step length (cm) and swing time (% of gait cycle) were calculated and expressed as symmetry ratios (paretic/non-paretic) at each time point. We then calculated symmetry deviation values at each time point as: *symmetry deviation* = |1 − *symmetry*|. Symmetry deviation values reflect how asymmetric the symmetry ratio was, regardless of direction of asymmetry (paretic>non-paretic or paretic<non-paretic). We calculated change in symmetry deviation between time points as: *symmetry* = |1 − *post symmetry*| − |1 − *pre symmetry*| and *symmetry* = |1 − *follow* − *up symmetry*| − |1 − *post symmetry*|. Negative values indicate improved symmetry between time points, and positive values indicate more asymmetry, regardless of the direction of change. For example, Δ symmetry would be −0.1 if symmetry ratio changed from 0.8 to 0.9, from 1.2 to 1.1, or from 0.8 to 1.1. Because this method of calculating change in symmetry does not capture the magnitude of change when values cross 1, we also calculated change in symmetry ratio between time points: *symmetry* = *post symmetry* − *pre symmetry*, and *symmetry* = *follow* − *up symmetry* − *post symmetry*. The sign of this metric does not tell you whether symmetry was increased or decreased, just the direction and magnitude in which the symmetry ratio changed. This is particularly relevant for step length symmetry, which had asymmetry in either direction in our sample.

### Statistical Analysis

As reported previously, change in walking speed after training did not differ between groups (17). Change in spatiotemporal symmetry also did not differ between groups. Therefore, for these secondary analyses, data were pooled irrespective of group. Symmetry ratios and symmetry deviation values were compared with perfect symmetry (value of 1 and 0, respectively) at each time point with one-sample *t*-tests. Walking speed, step length, swing time, symmetry ratios, and symmetry deviation values were compared between pre- and post-assessment and 3-month follow-up with a repeated measures ANOVA (within subject factor of testing session). For non-spherical data (Mauchly's Test), the Greenhouse-Geisser correction was used. *Post-hoc* pairwise comparisons between sessions were performed with Bonferroni correction. Cross-sectional associations of walking speed with symmetry ratios and symmetry deviation values and associations of change in walking speed with change in symmetry ratios and symmetry deviation values were tested with Pearson correlations. All statistical analyses were performed with SPSS Statistics 25 (IBM, NY, USA), with two-sided statistical testing with an alpha of 0.05.

## Results

All 81 participants were included in baseline correlations (demographics in Table 1). Five participants were lost to follow-up at the post-assessment, 4 were lost to follow-up at 3-month follow-up, and data from 1 participant were excluded because they had an increase in walking speed that was >4 SD above the mean. Thus, analyses of walking speed changes were performed on a subject pool of 71. For spatiotemporal symmetry, limited data from individual participants were also excluded if >4 SD above/below the mean.

**Table 1:** Demographics.

	**Total (*n* = 81)**
Age [years, mean (SD)]	58.7 (9.4)
Sex (male/female, counts)	55/26
More affected limb (left/right, counts)	44/37
Years since stroke [mean (Range)]	5.5 (0.5–21.8)
Type of stroke (ischemic/hemorrhagic, counts)	53/26^*^
Fugl-Meyer lower extremity	
Motor score [mean (SD)]	21.1 (4.3)
Sensory score [mean (SD)]	10.4 (2.7)

*Values assessed at pre-assessment*.

* *Information on stroke type was unavailable for two participants*.

**Figure 1:**
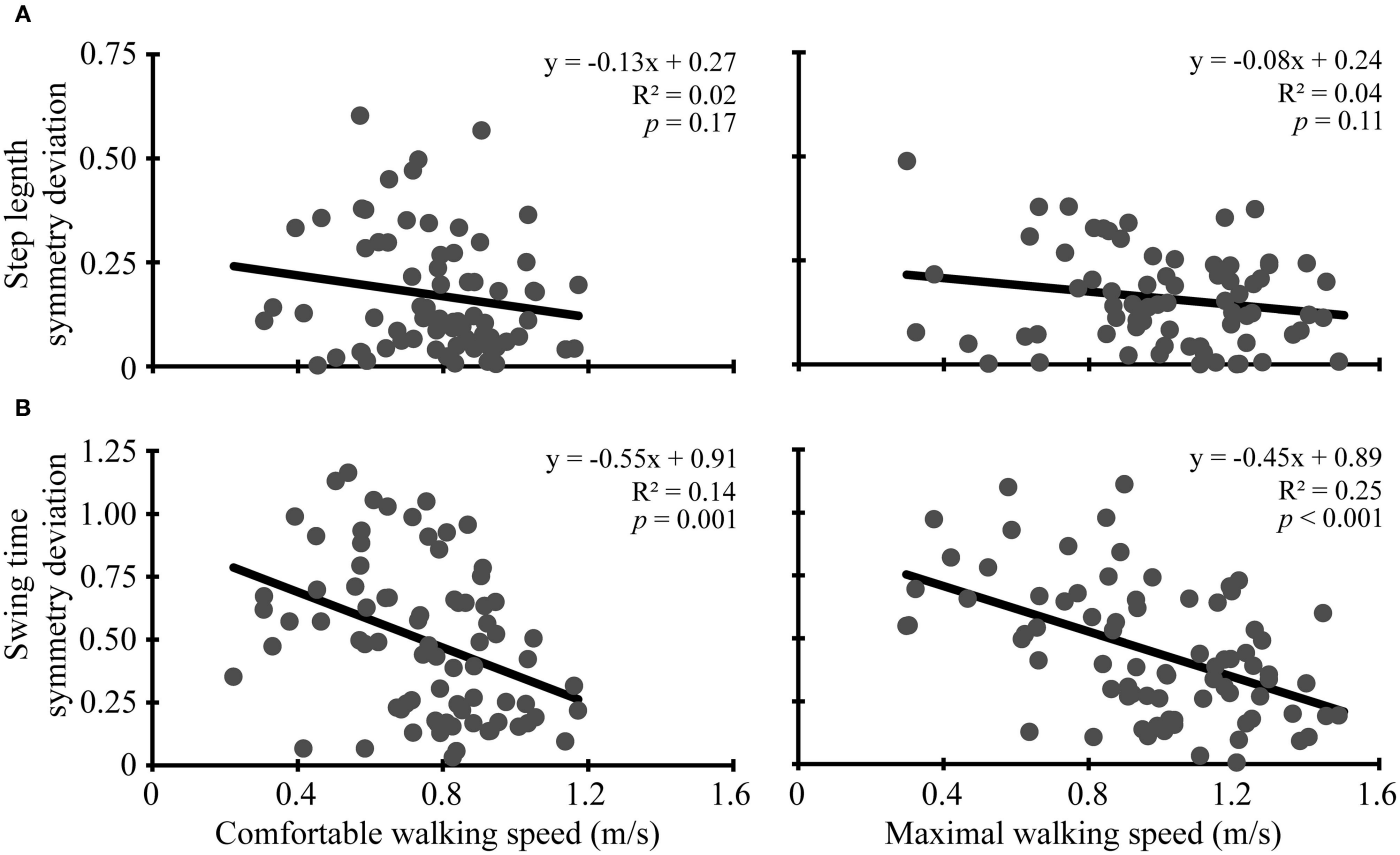
Baseline correlations. Scatter plots with lines of best fit show the association between comfortable (left column) and maximal (right column) walking speeds and **(A)** step length symmetry deviation and **(B)** swing time symmetry deviation. Symmetry deviation was calculated as: |1-(paretic/non-paretic)|. Gray dots represent data from individual participants. Black lines are lines of best fit with the equation, *R*^2^ value, and *p*-value displayed.

At pre-assessment, step length ratios deviated from symmetry during walking at comfortable (mean symmetry deviation: 0.17, 95% CI: 0.14, 0.21, *t* = 9.9, *p* < 0.001) and maximal speeds (mean symmetry deviation: 0.16, 95% CI: 0.13, 0.18, *t* = 11.8, *p* < 0.001). Similarly, at pre-assessment, swing time ratios deviated from symmetry during walking at comfortable (mean symmetry deviation: 0.50, 95% CI: 0.43, 0.56, *t* = 14.2, *p* < 0.001) and maximal speeds (mean symmetry deviation: 0.45, 95% CI: 0.39, 0.51, *t* = 14.9, *p* < 0.001). Like symmetry deviation, symmetry ratios also were asymmetrical at pre-assessment for step length (*t* ≥ 5.0, *p* < 0.001) and swing time (*t* ≥ 14.2, *p* < 0.001). Symmetry deviation and symmetry ratios still indicated asymmetry at post-assessment (*t* ≥ 4.2, *p* < 0.001) and 3-month follow-up (*t* ≥ 5.2, *p* < 0.001). More symmetrical swing time (R^2^ ≥ 0.14, *p* ≤ 0.001) was correlated with faster comfortable and maximal walking speed. Step length symmetry deviation was not correlated with comfortable or maximal walking speed (R^2^ ≤ 0.04, *p* ≥ 0.11) (see Figure 1).

Comfortable and maximal walking speed varied with testing session (*p* < 0.001; Table 2). Comfortable walking speed was greater at post-assessment (mean difference: 0.08, 95% CI: 0.05, 0.12, *p* < 0.001) and follow-up (mean difference: 0.05, 95% CI: 0.02, 0.09, *p* = 0.002) than at pre-assessment, but not different from post-assessment to follow-up (mean difference: −0.03, 95% CI: −0.06, 0.003, *p* = 0.10). Maximal walking speed was greater at post-assessment (mean difference: 0.12, 95% CI: 0.09, 0.16, *p* < 0.001) and follow-up (mean difference: 0.08, 95% CI: 0.04, 0.12, *p* < 0.001) than at pre-assessment, and greater at post-assessment than at follow-up (mean difference: 0.04, 95% CI: 0.02, 0.07, *p* = 0.001). Changes in step length and swing time values, symmetry ratios, and symmetry deviation values are shown in Table 2. There was no change in symmetry deviation for step length (*p* ≥ 0.15) or swing time (*p* ≥ 0.14) at comfortable or maximal walking speeds. There also was no change in symmetry ratio for step length (*p* ≥ 0.10) or swing time (*p* ≥ 0.14) at comfortable or maximal walking speeds.

**Table 2:** Walking speed and spatiotemporal symmetry.

			**Pre**	**Post**	**3M**	**Post-Pre**	**3M-Pre**	**3M-Post**	***F***	***p***
Speed (m/s)		Comfortable	0.76 (0.22)	0.84 (0.22)^*^	0.81 (0.24)^*^	0.08 (0.12)	0.05 (0.12)	−0.03 (0.11)	16.5	**<0.001**
		Maximal	0.98 (0.30)	1.11 (0.33)^*^	1.06 (0.33)^*^^†^	0.12 (0.12)	0.08 (0.13)	−0.04 (0.11)	36.1	**<0.001**
Step length (cm)	Paretic	Comfortable	50.8 (9.5)	54.2 (10.4)^*^	52.7 (9.8)^*^^†^	3.4 (5.5)	1.9 (5.6)	1.5 (4.9)	11.9	**<0.001**
		Maximal	59.3 (11.2)	62.5 (11.7)^*^	61.7 (11.9)^*^	3.2 (4.0)	2.5 (5.9)	−0.7 (5.3)	13.2	**<0.001**
	Non-paretic	Comfortable	45.5 (9.2)	49.3 (9.6)^*^	47.4 (10.2)^*^^†^	3.8 (4.9)	1.9 (5.3)	−1.9 (5.1)	18.2	**<0.001**
		Maximal	54.1 (10.9)	58.0 (11.2)^*^	55.7 (12.4)^†^	4.0 (5.0)	1.6 (5.8)	−2.4 (5.8)	19.8	**<0.001**
	Ratio (paretic/ non-paretic)	Comfortable	1.12 (0.19)	1.10 (0.19)	1.12 (0.19)	−0.02 (0.12)	−0.002 (0.12)	0.02 (0.13)	1.0	0.39
		Maximal	1.10 (0.16)	1.09 (0.17)	1.12 (0.19)	−0.008 (0.11)	0.02 (0.13)	0.03 (0.10)	2.4	0.10
	Symmetry deviation	Comfortable	0.17 (0.15)	0.15 (0.15)	0.17 (0.14)	−0.02 (0.12)	−0.007 (0.09)	0.01 (0.11)	0.9	0.41
		Maximal	0.15 (0.11)	0.15 (0.12)	0.17 (0.14)	−0.004 (0.10)	0.02 (0.12)	0.02 (0.09)	2.0	0.15
Swing time (%GC)	Paretic	Comfortable	35.8 (4.2)	37.4 (4.1)^*^	37.2 (4.1)^*^	1.6 (3.4)	1.4 (3.3)	−0.2 (2.6)	7.8	**0.001**
		Maximal	39.0 (4.0)	39.9 (4.0)^*^	39.1 (4.5)	0.9 (2.2)	0.1 (2.9)	−0.8 (2.8)	4.6	**0.02**
	Non-paretic	Comfortable	25.0 (4.5)	25.9 (4.1)^*^	25.5 (4.5)	0.9 (2.9)	0.5 (2.9)	−0.4 (2.9)	3.5	**0.04**
		Maximal	27.5 (4.1)	28.8 (4.4)^*^	28.1 (4.6)	1.3 (1.7)	0.6 (2.2)	−0.7 (2.5)	11.9	**<0.001**
	Ratio (paretic/ non-paretic)	Comfortable	1.48 (0.31)	1.48 (0.30)	1.51 (0.32)	0.002 (0.14)	0.03 (0.16)	0.03 (0.13)	1.0	0.35
		Maximal	1.45 (0.26)	1.42 (0.27)	1.43 (0.27)	−0.03 (0.12)	−0.02 (0.12)	0.01 (0.13)	2.0	0.14
	Symmetry deviation	Comfortable	0.48 (0.31)	0.48 (0.30)	0.51 (0.32)	0.002 (0.14)	0.03 (0.16)	0.03 (0.13)	1.0	0.35
		Maximal	0.45 (0.26)	0.42 (0.27)	0.43 (0.27)	−0.03 (0.12)	−0.02 (0.12)	0.01 (0.13)	2.0	0.14

*Comfortable and maximal 10-meter walk test speeds, step length, swing time, symmetry ratios, and symmetry deviation values for step length and swing time are shown for the pre-assessment (Pre), post-assessment (Post), and 3-month follow-up (3M). Also shown are differences between testing sessions. F and p-values represent the results from each ANOVA. Statistical significance symbols represent the results from post-hoc pairwise comparisons*.

* *p < 0.05 compared to pre-assessment*.

† *p < 0.05 compared to post-assessment*.

*Bold values represent statistically significant p-values*.

**Figure 2:**
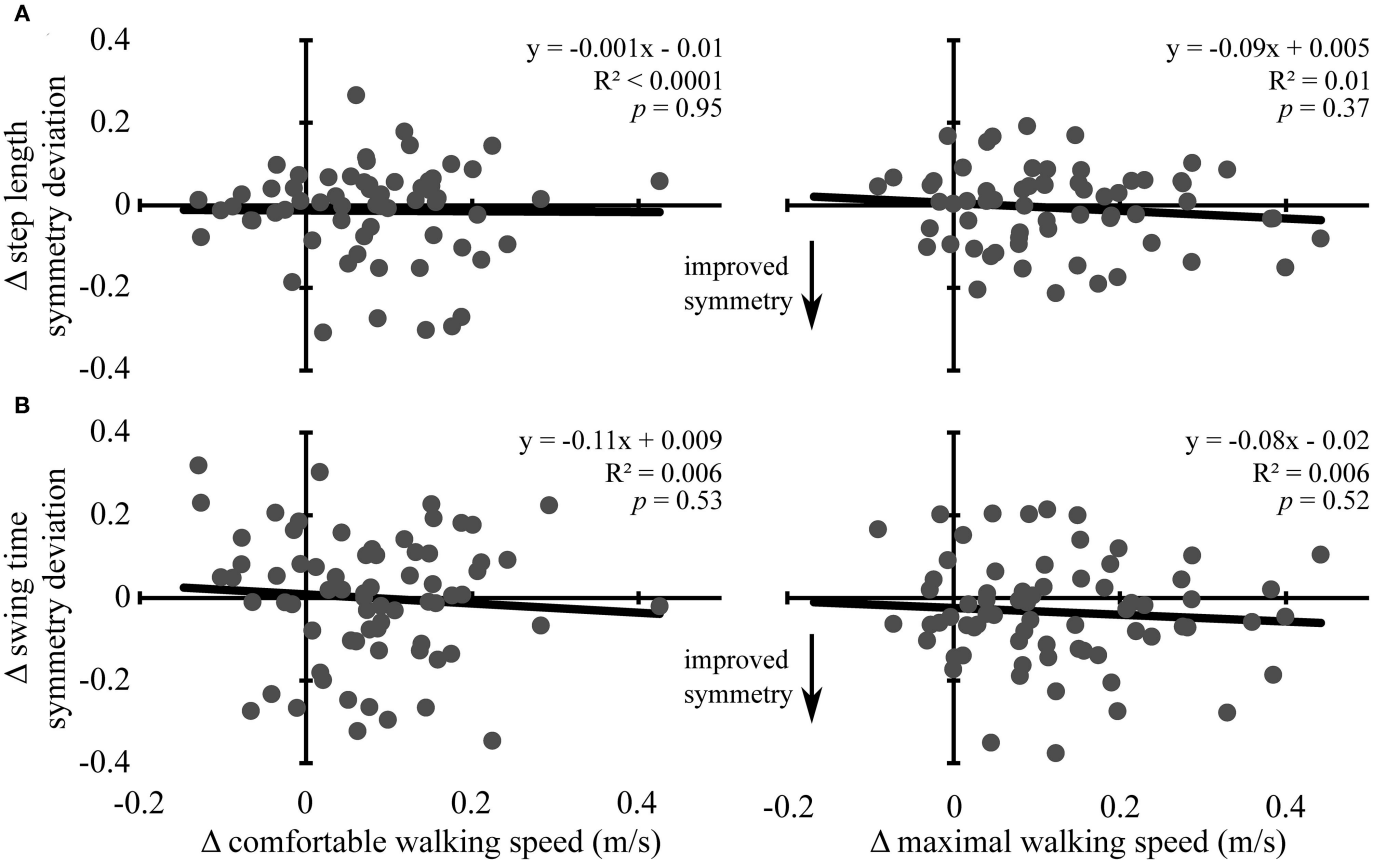
Change in walking speed and symmetry: pre- to post-assessment. Scatter plots with lines of best fit show the association between change in comfortable (left column) and maximal (right column) walking speeds and change in **(A)** step length symmetry deviation and **(B)** swing time symmetry deviation from pre-assessment to post-assessment. Change in symmetry deviation was calculated as: *symmetry* = |1 − *post symmetry*| − |1 − *pre symmetry*|. Negative values indicate improved symmetry between time points, and positive values indicate more asymmetry, regardless of the direction of change. Gray dots represent data from individual participants. Black lines are lines of best fit with the equation, *R*^2^ value, and *p*-value displayed.

**Figure 3:**
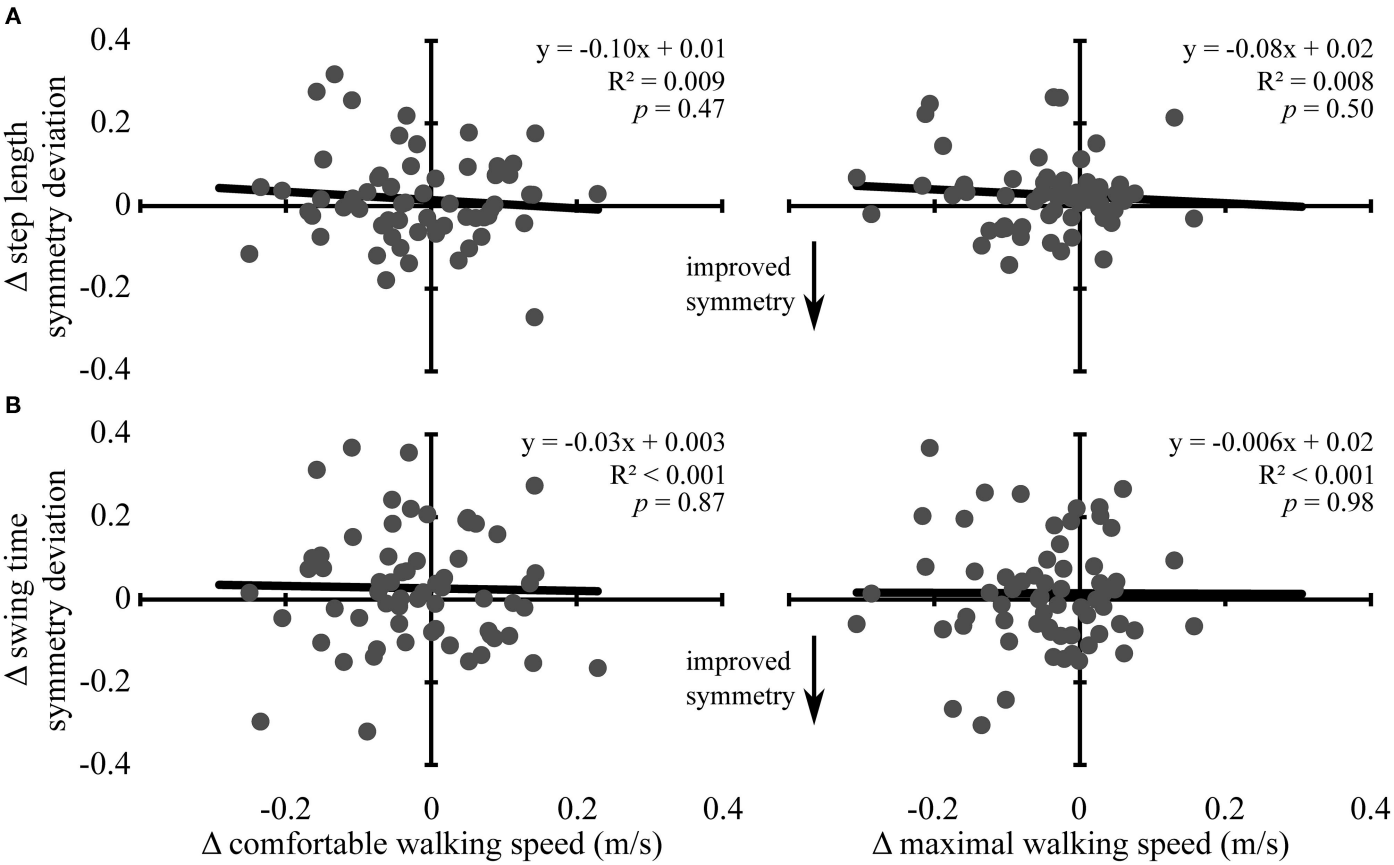
Change in walking speed and symmetry: post-assessment to 3-month follow-up. Scatter plots with lines of best fit show the association between change in comfortable (left column) and maximal (right column) walking speeds and change in **(A)** step length symmetry deviation and **(B)** swing time symmetry deviation from post-assessment to 3-month follow-up. Change in symmetry deviation was calculated as: *symmetry* = |1 − *follow* − *up symmetry*| − |1 − *post symmetry*|. Negative values indicate improved symmetry between time points, and positive values indicate more asymmetry, regardless of the direction of change. Gray dots represent data from individual participants. Black lines are lines of best fit with the equation, *R*^2^ value, and *p*-value displayed.

Changes in comfortable and maximal walking speeds from pre- to post-assessment were not associated with changes in symmetry deviation for step length (R^2^ ≤ 0.01, *p* ≥ 0.37) or swing time (R^2^ ≤ 0.006, *p* ≥ 0.52) (see Figure 2). Similarly, change in comfortable and maximal walking speeds from post-assessment to follow-up were not associated with change in symmetry deviation for step length (R^2^ ≤ 0.009, *p* ≥ 0.47) or swing time (R^2^ < 0.001, *p* ≥ 0.87) (see Figure 3). No significant relations were found between change in walking speed and change in symmetry ratio (R^2^ ≤ 0.04, *p* ≥ 0.12). Furthermore, changes in symmetry deviation were not associated with baseline walking speed (R^2^ ≤ 0.05, *p* ≥ 0.09).

To account for issues with pooling data across the four experimental groups in this study, we also performed associative analyses between change in walking speed, step length, and swing time separately for each of the four experimental groups. There were no significant associations for any of the groups. Additionally, this study included some participants who were relatively symmetric, which could have impacted our findings. To address this issue, we performed a sub-analysis only using individuals who were asymmetric at baseline. Individuals with step length symmetry ratios between 0.9 and 1.1 were considered symmetric (8), and individuals with ratios above or below this range were considered asymmetric (*n* = 42 for comfortable speed and *n* = 47 for maximal speed). In this subset, we found that, at baseline, greater symmetry deviation was associated with slower maximal walking speed (R^2^ = 0.22, *p* = 0.001). Change in symmetry deviation was not correlated with change in either comfortable or maximal walking speed from pre- to post-assessment (R^2^ ≤ 0.02, *p* ≥ 0.33) or from post-assessment to follow-up (R^2^ ≤ 0.03, *p* ≥ 0.32). We did not perform a sub-analysis for swing time symmetry ratio because only 7% (*n* = 6) of our participants had ratios <1.1 for at least one walking speed.

## Discussion

In this study in individuals with chronic stroke, we assessed the association between walking speed and spatiotemporal symmetry, whether HISTT leads to changes in spatiotemporal symmetry, and whether changes in walking speed following HISTT are associated with changes in symmetry. Cross-sectionally, we found that individuals with faster walking speeds had more symmetrical swing time. However, although HISTT-related improvements in walking speed were observed, HISTT did not affect walking symmetry, and changes in walking speed were not associated with changes in symmetry. These results have implications for future rehabilitative efforts.

Cross-sectionally, we found that stroke survivors had swing time asymmetry, and that greater asymmetry was associated (albeit weakly) with slower walking speeds, as previously shown (3, 5–8, 10, 11). Although the cross-sectional relation between swing time symmetry and walking speed may reflect a causative relation, our results from training with HISTT (discussed below) suggest that the relation between swing time symmetry and walking speed may be mediated by other factors, such as stroke-related impairments in muscle strength, coordination, or sensation or the extent of damage to descending motor tracts.

In contrast to swing time symmetry (temporal), step length symmetry (spatial) was asymmetric but not associated with walking speed in the current study. Previous studies have been ambivalent about the relation between step length symmetry and walking speed, with some studies showing no relation (4, 7, 8) but others showing a significant relation (10, 11, 14). The variability of findings across studies may reflect that the direction of step length asymmetry is variable, and fewer individuals present with asymmetry of this outcome measure. In the current study, 27% of our participants had symmetry values <1, while 73% had symmetry values >1 at the pre-assessment, consistent with data from other studies (7, 8, 10, 14). In contrast, 100% of participants had longer paretic than non-paretic swing time. Our sub-analysis including only relatively asymmetric individuals found a significant relation between step length symmetry and maximal walking speed. Furthermore, 39 participants (48%) had relatively symmetric step length symmetry ratios (8) at comfortable speed (0.9–1.1), while only 6 participants (7%) had relatively symmetric swing time symmetry ratios. Our results suggest that step length asymmetry (spatial) is of a lower prevalence and magnitude than swing time asymmetry (temporal). Also, disparities between spatial and temporal symmetry suggest that these measures reflect different aspects of walking (27, 28).

After 4 weeks of HISTT both comfortable and maximal walking speeds increased and remained elevated 3 months later (17). We expected to also detect improvements in spatiotemporal symmetry that were related to these improvements in walking speed because some studies have shown simultaneous improvements in speed and symmetry (although direct relations were not tested) in response to a variety of moderate-intensity walking training interventions (29–35). To the contrary, we did not find significant changes in either step length (spatial) or swing time (temporal) symmetry, and changes in symmetry/symmetry deviation were not associated with changes in walking speed. These findings were true for the training period (pre- to post-assessment) and for the retention period (post-assessment to 3-month follow-up). Our results suggest that HISTT does not improve or worsen spatiotemporal symmetry and that walking speed can improve and be retained regardless of changes in symmetry. These findings do not appear to be related to the high-intensities involved in HISTT because other studies using a variety of moderate-intensity training interventions have also found improvements in walking speed with no change in symmetry (although direct relations were not tested) (36–40). Only a couple studies have reported the direct relation between change in walking speed and spatiotemporal symmetry; one found that increased propulsive *asymmetry* was associated with greater improvements in walking speed (39), while another (41) found no relation between changes in walking speed and changes in spatiotemporal symmetry.

Considered together, these results suggest that, although there is a cross-sectional relation between walking speed and swing time (temporal) symmetry, improvements in walking speed induced by walking rehabilitation may be independent of improvements in spatiotemporal symmetry. Divergence between walking symmetry and speed may occur because walking speed can be increased through a variety of different strategies. Some individuals may improve walking speed through increased spatiotemporal symmetry, while others may improve walking speed by accentuating asymmetries (1, 19). In the current study, a slight minority of individuals who improved comfortable walking speed from pre- to post-assessment also improved step length (43%) and swing time symmetry (49%). Although symmetry is often assumed to yield faster walking speeds, some asymmetries are actually associated with faster walking (42, 43). Common compensatory strategies that may enhance walking speed include increased non-paretic limb propulsion, paretic hip circumduction, paretic knee hyperextension, and bilateral hip flexion (43–45). Overall, our results suggest that restoring symmetry may not be necessary for improving walking speed with rehabilitation after stroke (1, 43).

These findings have important implications for walking rehabilitation after stroke. If the goal of rehabilitation is to improve walking speed, then interventions like HISTT can achieve this result without a systematic effect on spatial or temporal walking symmetry. The mean changes in comfortable (0.08 m/s) and maximal (0.12 m/s) walking speed are likely to represent small to substantially meaningful changes (46). However, some evidence suggests that walking asymmetry may limit recovery in the paretic limb, impair dynamic balance, decrease movement efficiency, and lead to long-term overuse injuries (19, 20). Consequently, clinicians may need to pair walking interventions like HISTT with another intervention designed to improve walking symmetry simultaneously. More traditional approaches to improving walking symmetry include therapist- or robotic-assisted training that guides spatiotemporal symmetry during walking (38). Additionally, unilateral step training with the non-paretic limb (47) and walking on a split-belt treadmill (48) can improve step length asymmetry. Other strategies during locomotor training include walking with acoustic guidance (49) or using functional electrical stimulation (50). Similarly, pedaling on a supine or recumbent bike with a split crankshaft increases muscle activation in the paretic limb and may be useful for training interlimb coordination and symmetry (51). Combining interventions such as these with HISTT may prove to be beneficial.

## Limitations

The results presented here are from secondary analyses of a randomized controlled trial which was not designed to directly assess the purpose of the current study. The original study randomly assigned participants to one of four experimental groups dictating a priming condition received prior to HISTT. In the current study, participants from all groups were assessed without regard to experimental group. Because there was no control group that did not receive HISTT, we cannot directly assess whether HISTT leads to changes in the walking speed or spatiotemporal symmetry. Furthermore, we can only estimate whether HISTT-induced changes in walking speed are associated with changes in spatiotemporal symmetry because participants were not randomly assigned to groups designed to test these questions, and the type of priming received may have impacted the results. Although we identified a significant cross-sectional relation between swing time symmetry and walking speed, it is important to note that the variance explained by this relation is small. Additionally, our correlations reveal some evidence of heteroscedasticity, suggesting that the cross-sectional relation of symmetry/symmetry deviation with walking speed and change in symmetry/symmetry deviation with change in walking speed may be inconsistent. Hence, the correlation coefficients and associated *p*-values should be interpreted cautiously. This study was conducted in individuals with chronic stroke, so results may differ in individuals with acute or sub-acute stroke.

## Data Availability Statement

The raw data supporting the conclusions of this article will be made available by the authors, without undue reservation.

## Ethics Statement

The studies involving human participants were reviewed and approved by University of Illinois at Chicago Institutional Review Board. The patients/participants provided their written informed consent to participate in this study.

## Author Contributions

BC and SM contributed to conception and design of the study, acquisition and analysis of data, drafting the manuscript, and figures. All authors contributed to the article and approved the submitted version.

## Conflict of Interest

The authors declare that the research was conducted in the absence of any commercial or financial relationships that could be construed as a potential conflict of interest.

## Abbreviations

AMT: ankle motor tracking
HISTT: High-intensity speed-based treadmill training
SD: standard deviation
tDCS: transcranial direct current stimulation.
